# ASO Author Reflections: Modelling Morbidity in Retroperitoneal Sarcoma—Multimodal Therapy is Safe While Surgery Sets the Tone

**DOI:** 10.1245/s10434-025-18386-3

**Published:** 2025-09-29

**Authors:** Mathilda Knoblauch, Markus Albertsmeier

**Affiliations:** Department of General, Visceral and Transplantation Surgery, Ludwig-Maximilians-Universität (LMU) Munich, LMU Hospital, Munich, Germany

## Past

Complete surgical resection remains the cornerstone of cure for retroperitoneal soft tissue sarcoma (RPS). To improve local control and survival, multimodal regimens, including radiotherapy, systemic chemotherapy, and, in a few specialized centers, regional hyperthermia (RHT), have been explored.^1^ Yet, their potential to increase perioperative morbidity remains a concern. This study assessed the impact of neoadjuvant treatment protocols, compared with surgical driven complications, on postoperative morbidity.^2^

## Present

In our single-center cohort of 335 RPS resections, neoadjuvant therapies were associated with higher complication rates in univariable analysis. However, in multivariable models, none of these treatments, including combinations with RHT, independently increased postoperative morbidity. Instead, the perioperative risk was determined by surgical factors, particularly multivisceral resections and transfusion requirements. The apparent association between neoadjuvant regimens and morbidity thus seems to reflect underlying disease aggressiveness rather than treatment-related toxicity. Methodologically, we employed the Comprehensive Complication Index (CCI),^3^ which provides a more nuanced measure than the Clavien-Dindo classification. To address its skewed distribution at zero, intermediate, and fatal values, a zero–one inflated beta regression was applied to model postoperative morbidity with greater precision.^4^ This approach has not been used in previous related studies.

## Future

Future studies should focus on strategies to reduce transfusion requirements, including prehabilitation, optimization of hemoglobin levels, and intraoperative blood-sparing techniques. Moreover, the analytical approach used here may also serve to evaluate the safety of novel neoadjuvant strategies, such as radioimmunotherapy or thermosensitive liposomes, which could soon complement standard regimens. Finally, CCI-based multivariable models should be applied in future studies across the oncologic field to standardize morbidity reporting and enable meaningful comparisons across tumor types.
